# Machine learning-based LASSO-Cox model for dementia prediction: The role of midlife cardiometabolic, inflammatory, and genetic risk factors in a US cohort

**DOI:** 10.1016/j.annepidem.2026.01.007

**Published:** 2026-01-23

**Authors:** Longjian Liu, Jintong Hou

**Affiliations:** Department of Epidemiology and Biostatistics, Dornsife School of Public Health, Drexel University, Philadelphia, PA, 19104, USA

**Keywords:** Machine Learning, LASSO-Cox Modeling, Midlife Features and Dementia Risk

## Abstract

**Purpose:**

We aimed to identify key midlife dementia predictors and develop a novel machine learning (ML)-enabled risk prediction model.

**Methods:**

Using data from 9266 Atherosclerosis Risk in Communities study participants (aged 45–64 years at baseline, 1987–1989). Incident dementia was ascertained through December 2019. A ML-based LASSO-Cox model was applied to develop the risk prediction model.

**Results:**

Over a 25-year mean follow-up, 2010 participants developed dementia. The LASSO-Cox model identified 12 key predictors and achieved C-indices (95 %CI) of 0.77 (0.75–0.79) in the training set (n = 6182) and 0.78 (0.76–0.81) in the test set (n = 3084). Predictors included age, Digit Symbol Substitution Test, apolipoprotein E ε4, HbA1c, brachial blood pressure, Factor VIII, Delayed Word Recall Test, hypertension, stroke history, C-reactive protein, white blood cell count, and apolipoprotein B. The resulting nomogram demonstrated strong discrimination (AUC 0.77–0.86) and good calibration. LASSO-Cox risk score quartiles effectively stratified participants into low, moderate, high, and very high dementia risk groups.

**Conclusions:**

The findings demonstrate that the newly developed machine learning-based LASSO-Cox model provides a robust method to predict individuals at high risk of dementia.

## Introduction

Dementia represents a growing public health challenge in the United States and globally, with the number of affected individuals expected to rise substantially as populations age [1,2]. Early identification of individuals at elevated risk is critical for implementing timely interventions that may delay the onset or progression of disease. However, dementia is a multifactorial condition influenced by a complex interplay of genetic, vascular, metabolic, inflammatory, and lifestyle factors, many of which interact and evolve across the life course [3–7]. Among known genetic risk factors, the apolipoprotein E (APOE) ε4 allele remains the strongest predictor of late-onset dementia. In addition to genetic predisposition, numerous studies, including our own, have identified cardiometabolic conditions such as hypertension, diabetes, and dyslipidemia as important and potentially modifiable contributors to cognitive decline and dementia [3,5–9]. Recent advances in artificial intelligence (AI) and machine learning (ML) offer promising opportunities to improve risk prediction by leveraging natural language processing, image recognition, high-dimensional data, and ML-based algorithms to detect complex, linear and nonlinear associations that traditional statistical approaches may overlook or underlook. AI/ML approaches can enhance the precision of early identification efforts and support more effective, personalized and population-based prevention strategies. In this study, we applied an integrated ML survival analysis framework that integrates penalized Cox regression with Least Absolute Shrinkage and Selection Operator (LASSO) analysis to identify key predictors of dementia risk. Using data from the well-characterized Atherosclerosis Risk in Communities (ARIC) Study, we examined 71 candidate variables across demographic, behavioral, cardiometabolic, renal, inflammatory, and genetic domains. Our goal was to develop and validate a ML–enabled prediction model capable of classifying middle-aged adults by their risk of developing dementia over a mean follow-up of 25 years.

## Methods

### Study population

We analyzed data from the Atherosclerosis Risk in Communities (ARIC) Study, a prospective cohort study designed to investigate the etiology and outcomes of cardiovascular and related chronic diseases in a biracial population. The baseline examination (Visit 1) included 15,792 participants aged 45–64 years recruited between 1987 and 1989 from four U.S. communities: Forsyth County, North Carolina; Jackson, Mississippi; suburban Minneapolis, Minnesota; and Washington County, Maryland. Measurements of cognitive function were performed in Visit 2 (1990–1992) in 14,348 participants from the original cohort that provided the baseline cognitive assessment for this report. This assessment was described elsewhere [10,11]. Incident dementia cases were identified annually through December 2019. The ARIC study protocol was approved by the institutional review boards (IRBs) at each participating center, and all participants provided written informed consent. We analyzed the ARIC Study data, obtained from the NHLBI’s Biologic Specimen and Data Repository Information Coordinating Center (BioLINCC; RMDA No. 13661) with IRB approval from Drexel University (No. 2308010042). Of the 14,348 participants, we excluded those with missing data on cognitive function tests or APOE genotyping (n = 1640); those with missing data for education attainment status, smoking status, measures of body mass index, blood pressure, and markers of cardiometabolic, chronic kidney disease, serum C-reactive protein and Cystatin C, and chronic conditions of diabetes mellitus, coronary heart disease, heart failure, and stroke (n = 3442). The final analytic sample size of the complete data included 9266 participants.

### Outcome ascertainment

#### Dementia Ascertainment:

Incident dementia cases were identified using a combination of neurocognitive testing, clinical adjudication, ICD-9 and ICD-10 hospital discharge codes, and death certificate codes. This diagnosis process was particularly enhanced through the ARIC Neurocognitive Study (ARIC-NCS), which began at Visit 5 (2011–2013). Adjudicated dementia cases were identified based on established criteria consistent with DSM-IV and NINCDS-ADRDA guidelines from in-person cognitive testing at ARIC–NCS [12,13]. Information available to adjudicators included data from longitudinal evidence of cognitive decline based on cognitive assessments from prior visits, complete neuropsychological battery at the ARIC-NCS visits, and informant interviews. Among participants who did not attend the ARIC-NCS clinic visits, the Telephone Instrument of Cognitive Status–Modified was used to determine cognitive status and an informant telephone interview was conducted to classify dementia cases [7,13,14]. Participants were followed from baseline until the first diagnosis of dementia, death, loss to follow-up, or end of follow-up year (December 2019), whichever came first.

### Predictors

#### Cognitive Assessment:

Measures of cognitive function were assessed using a set of standardized batteries of three neurocognitive tests: (1) Digit Symbol Substitution Test (DSST): Assesses processing speed and executive function. (2) Delayed Word Recall Test (DWRT): Evaluates verbal memory. (3) Word Fluency Test (WFT): Measures verbal fluency and executive function [11]. We used z scores of DSST, DWRT, and WFT, provided by ARIC, which were calculated for each participant as the difference between the observed factor scores and the estimated factor scores derived from the robust normative sample divided by the root mean squared error from the race-stratified regression model. This z-score transformation adjusted for baseline population differences and placed all participants on a common scale, thereby enabling direct comparisons of cognitive function across race groups [12–16]. Higher *z* scores denote better than expected performance on domain-specific cognitive tests, while lower *z* scores indicate the opposite. In LASSO models, these standardized scores were entered directly as continuous predictors in a single LASSO-Cox model. This model also included socio-demographic factors to control for potential confounding. This approach served a dual purpose: it reduced bias due to baseline demographic differences while allowing us to utilize the maximum sample size for increased statistical power, thereby avoiding the need to develop separate, sub-specific prediction models.

#### Baseline demographic factors and clinical assessments:

Age (years), race and ethnicity (Non-Hispanic Black/African Americans [AA] and Whites), educational attainment (<high school, high school graduate or high school equivalent or vocational school, and college or above), smoking status (current, former, and never), alcohol consumption, physical activity, body mass index (BMI, calculated as weight [Kg] divided by height-square [m^2^]), blood pressure (BP), ankle and brachial SBPs (systolic BPs). Several serum biomarkers were collected at baseline (visit 1 in 1987–1989) or part of biomarkers were collected in visit 2 (1990–1992), if they did not collect in visit 1 (e.g., C-reactive protein, Cystatin C etc., see Supplementary Table 1). The main biomarkers include lipids (total cholesterol, low and high density lipoprotein cholesterols, triglycerides), glycemic profiles (fasting glucose and HbA1c), serum high-sensitivity C-reactive protein (CRP, mg/L, a biomarker of inflammation), Cystatin C (mg/L, a biomarker of chronic kidney disease), white blood cell (WBC) count, and serum concentrations of fibrinogen, Factor VIII, Apoprotein A-I (apo A-I), Apo B. Supplementary Tabel 1 (S-Table 1) shows the full list of these candidate predictors (n = 71) used in LASSO-Cox model to select feature importance. All blood samples were collected at baseline under standardized protocols and analyzed at ARIC central laboratories. Prevalent chronic conditions were assessed based on participants’ self-reported physician diagnoses of conditions, including hypertension, diabetes mellitus, coronary heart disease, heart failure, and stroke. APOE genotyping was performed using TaqMan assays on DNA extracted from stored blood samples. Participants were categorized based on the copy number of APOE ε4 allele (0, 1, or 2 copies). In LASSO-Cox and nomogram analysis, APOE ε4 status was classified as a binary variable (≥ 1 copy vs. none) to ensure stable hazard ratio estimates and adequate statistical power because the number of participants carrying two APOE ε4 alleles was small.

### Statistical analysis and machine-learning approach

In the first set of analysis, we described the baseline characteristics of participants by the study outcome, the incident dementia status. Differences in continuous variables were examined using Student’s *t*-tests, while differences in categorical variables were assessed using Chi-square tests.

**Fig. 1.**
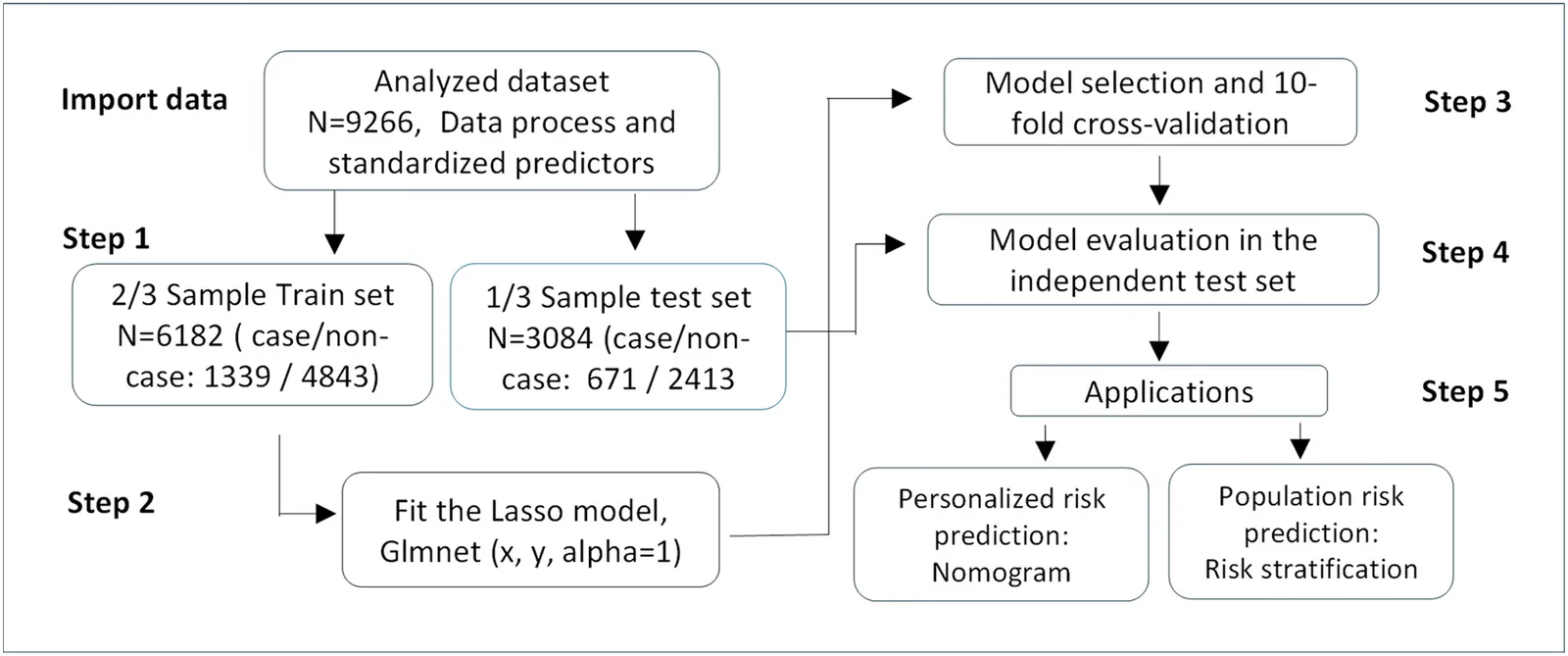
Workflow for the Development and Validation of a Machine Learning – Based Prediction Model.

In the second set of analysis, we performed Least Absolute Shrinkage and Selection Operator (LASSO) integrated with penalized Cox proportional hazards regression (LASSO-Cox) analysis to identify the most predictive features [17]. This approach is highly effective in avoiding redundancy or overfitting during feature selection [17–20]. Figure 1 depicts the analysis framework, which involved five integrated steps. Step 1: The total analysis dataset (N = 9266) was partitioned into a train set (n = 6182, two-thirds) and a test set (n = 3084, one-third). To ensure approximately equal proportions of dementia cases in both subsets, we employed stratified sampling based on the distribution of cases in the total sample. Step 2: We included a total of 71 candidate factors (Supplementary Table 1) in LASSO-Cox regression analysis to identify important independent features that predict dementia risk. The LASSO procedure was then applied, wherein the coefficients of the respective variables were progressively shrunk towards zero as the penalty factor (lambda, λ) increases. In cross-validation processes, it resulted in two commonly used lambdas: (a) λ_min_, the value of λ that minimizes the cross-validated error, typically selecting more variables but applies less regularization; and (b) λ_1se_, the largest λ within one standard error (1se) of the minimum error, which is more conservative, shrinking more coefficients to zero and resulting in fewer variables with stronger regularization. In this report, we used λ_1se_ for feature selection. Compared with λ_min_, λ_1se_ yields a more interpretable model with lower variance and better generalization to new data. All input variables were standardized (scaled) before applying LASSO to ensure equal penalization across predictors, prevent scale-driven bias in feature selection, and improve numerical stability. Step 3: The LASSO-Cox model was developed using the train dataset and evaluated internally using 10-fold cross-validation to prevent overfitting. λ_1se_ (0.02086873) was specified as the tunning parameter. A standard Cox model using LASSO selected predictors was developed using the unstandardized variables in the train data to make prediction. Step 4: Model performance was evaluated using the test dataset. To assess predictive accuracy, both the concordance index (C-index) and time-dependent area under the receiver operating characteristic curve (AUC) were calculated for the train and test datasets. The C-index had a value between 0.5 and 1, with 0.5 indicating that the model is completely random and 1 indicating that the model had perfect predictivity. A C-index above 0.70 is generally desirable and indicates meaningful predictive ability, specifically analyzing data from observational population-based research [21–23]. Step 5: A nomogram was derived based on the final selected important features from LASSO-Cox models to develop a novel pictorial representation of a complex mathematical formula [20,24–26]. The AUC plots were drawn by calculating the sensitivity (i.e., true positive rate) and specificity (true negative rate) for two follow-up timepoints at 10- and 20-year. To evaluate the nomogram’s predictive accuracy, we further created calibration plots for both train and test datasets according to a ten-fold cross-validation and 1000 bootstrap resamples to ensure robust predictions. The developed nomogram can be applied to predict dementia risk at individual levels. R package “RMS” was used to produce calibration plots and the nomogram. LASSO-Cox risk scores were categorized into quantiles for classifying participants into four risk levels from low, medium, high, to very high level.

To evaluate the robustness of our primary LASSO feature selection approach and to assess its predictive utility, we performed two sensitivity analyses (SA): SA-Elastic and SA-Cox. SA-Elastic (Stability Analysis) utilized Elastic Net regression on the training dataset to evaluate the feature stability across a range of mixing parameters (α = 0–1 in increments of 0.1), identifying the optimal α via 10-fold cross-validation with the λ_1se_ criterion. This analysis aimed to confirm whether the identified features remained stable across a spectrum of combined L1 (LASSO) and L2 (Ridge) penalties. SA-Cox (Predictive Comparison) compared the predictive performance and parsimony of the sparse LASSO model (12 predictors) against a full, non-penalized Cox proportional hazards model containing all 71 candidate predictors. Model discrimination was quantified using Harrell’s Concordance Index (C-index). By comparing C-indices and their associated 95 % confidence intervals across both training and test datasets, we determined if the LASSO approach, through simultaneous variable selection and shrinkage, provided measurable gains in predictive accuracy or enhanced model interpretability.

Statistical analyses were performed using SAS, version 9.4 (SAS Institute Inc., Cary, NC), and LASSO prediction modeling was performed using R version 4.2.2 of the Glmnet and Survival packages (https://www.r-project.org/). A 2-sided P-value of < 0.05 is regarded as statistically significant.

## Results

### Baseline Characteristics of the Participants and Incidence of Dementia

Table 1 shows that subjects with incident dementia over a mean of 25 years follow-up had higher baseline mean age, elevated body mass index (BMI), systolic blood pressure (SBP), diastolic BP, ankle and brachial SBP, total cholesterol, high density lipoprotein cholesterol (HDL), low density lipoprotein cholesterol (LDL), triglycerides, apolipoprotein A-I (Apo A-I), Apo B, and had lower cognition function measures of global, DSST, DWRT and WFT z scores than those without developed dementia. Women, African Americans, individuals with lower education levels, and those without health insurance had significantly higher risks of dementia compared with their respective counterparts. Subjects with dementia had a lower proportion of those with current smoking and alcohol consumption than those without dementia, and subjects with baseline chronic conditions of hypertension and stroke had higher rates of dementia, but a lower rate of coronary heart disease than those without dementia.

### LASSO-Cox modeling

Using LASSO-Cox regression, a comprehensive set of 71 variables (Supplementary Table 1) was analyzed to identify key predictors of dementia risk. In LASSO feature importance selection, lambda (λ) controls the penalty on coefficients. Twelve predictors (n = 12) were selected at λ_1se._

**Fig. 2.**
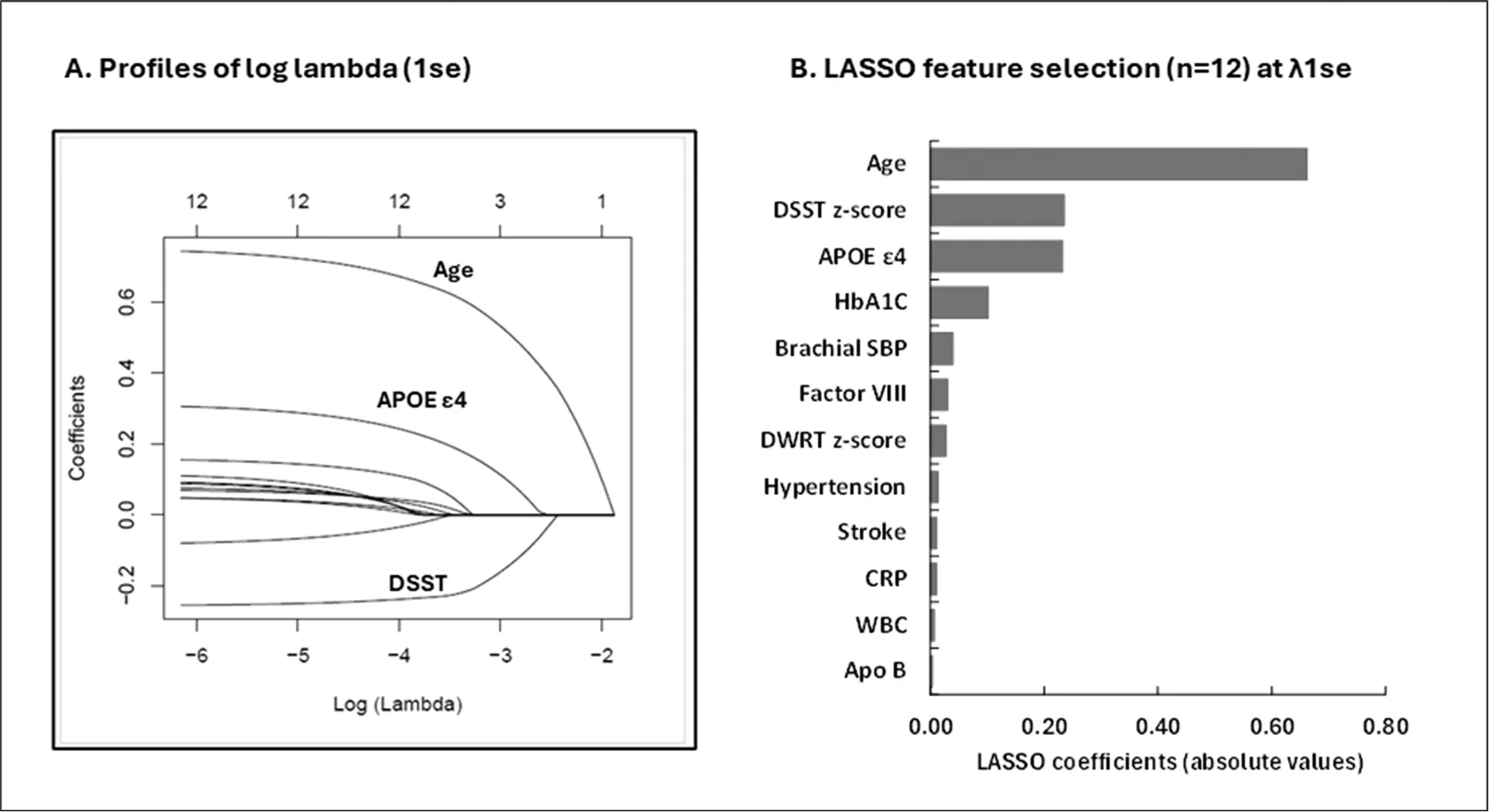
LASSO Identified Important Features. (A) Profiles of Log Lambda. (B) Ranks of the Identified Features by Absolute LASSO Coefficient Values. Note: DSST: Digit Symbol Substitution Test. APOE ε4: apolipoprotein E ε4. HbA1c: Hemoglobin A1c. SBP: Systolic blood pressure. DWRT: Delayed Word Recall Test. CRP: C-Reactive Protein. WBC: White Blood Cell. Apo B: Apolipoprotein B.

Figure 2A shows the LASSO coefficient profiles for dementia risk predictors. At the λ_1se_ threshold, 12 predictors remained non-zero, with age, DSST z-score, and APOE ε4 showing the strongest associations. Figure 2B depicts their relative importance, with age most influential (coefficient = 0.662), followed by DSST z-score, APOE ε4, HbA1c, blood pressure, Factor VIII, DWRT z-score, hypertension, stroke, CRP, white blood cell count, and apolipoprotein B. The LASSO-Cox model demonstrated robust predictive performance, with a cross-validated C-index of 0.752 and final C-indices of 0.771 (95 % CI: 0.754–0.787) in the training set and 0.783 (95 % CI: 0.760–0.806) in the test set.

Table 2 presents the LASSO coefficients (βs) and Cox hazard ratios (HRs) for the 12 key features selected by the LASSO-Cox model, all of which were associated with the risk of dementia. This analysis is based on a multivariate model where the effect of each selected predictor is mutually adjusted for the presence of all other factors in the table.

The magnitude and direction of the HRs confirm the relevance and distinct contribution of these features to the final sparse model. Interpretation of the top associations reveals the following: (1) Age: Each one-year increase in age was associated with a 14 % higher hazard of dementia (HR=1.14). (2) Cognitive measures: A one–standard deviation increase in DSST z-score was associated with a 24 % lower hazard of dementia (HR=0.76). (3) Genetics: Carriers of the APOE ε4 demonstrated a significantly higher hazard, showing an 89 % increased hazard of dementia (HR=1.89) compared with non-carriers. (4) Diabetes marker: Each 1 % absolute increase in serum HbA1c concentration was associated with a 13 % higher hazard of dementia. These selected predictors align with known risk factors in the existing literature [8–12,27].

### Nomogram establishment and validation

**Fig. 3.**
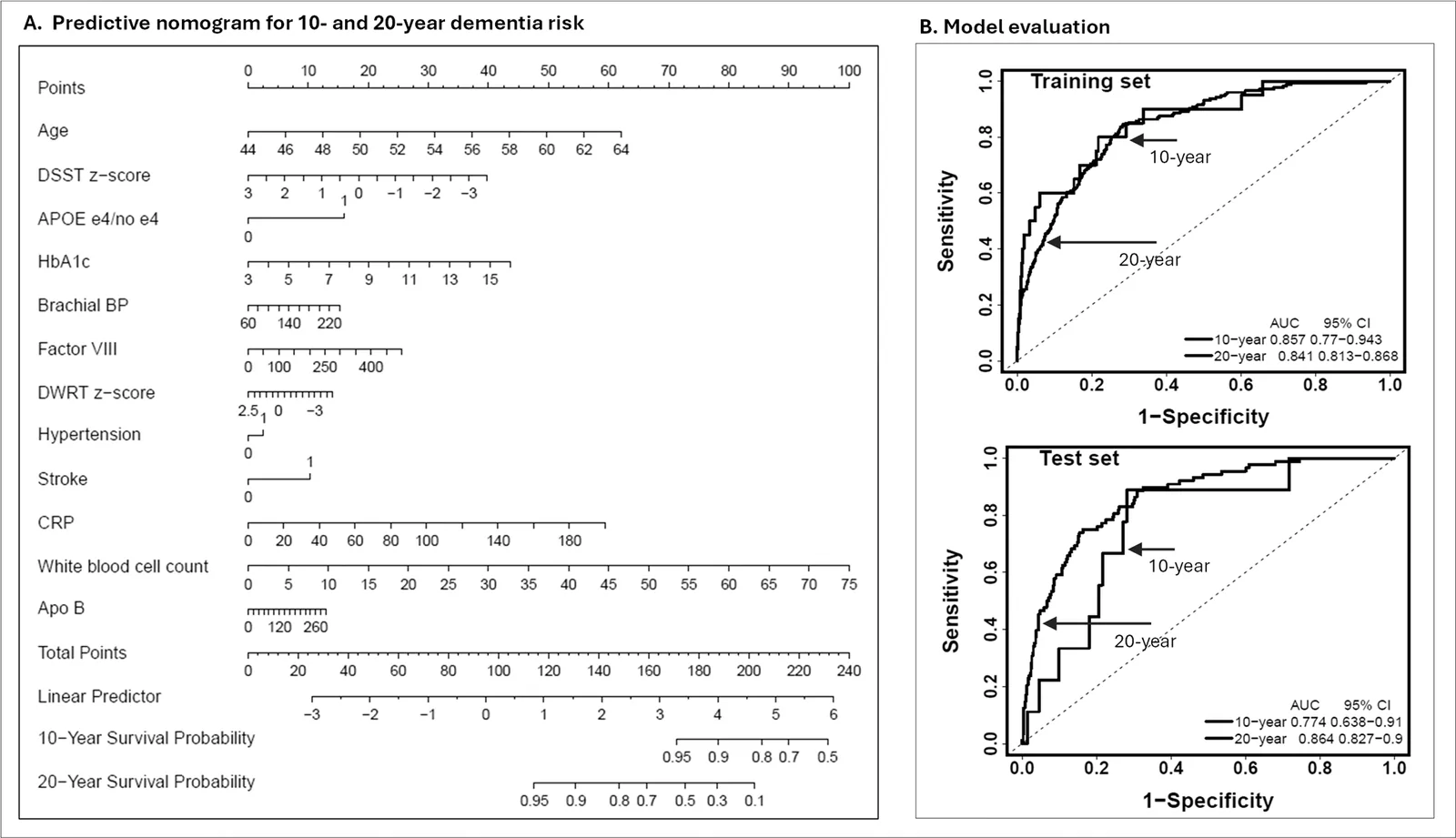
Predictive Nomogram for 10- and 20-Year Dementia Risk and Model Evaluation. Note: (A) The nomogram calculates a total risk score for predicting 10- and 20-year dementia risk by summing points assigned to each variable. (B) Model evaluation is presented using Area Under the Receiver Operating Characteristic (AUC) curves in training (B1) and test (B2) datasets. DSST: Digit Symbol Substitution Test. APOE ε4: Apolipoprotein E ε4. HbA1c: Hemoglobin A1c. BP: Blood pressure. DWRT: Delayed Word Recall Test. CRP: C-Reactive Protein. WBC: White Blood Cell. Apo B: Apolipoprotein B.

We developed a nomogram based on the 12 identified features to serve as a predictive tool for patient survival (Figure 3A). This nomogram was designed to allow for the calculation of total points by adding up the contributions of each variable. These final scores are then directly used to estimate an individual’s 10- and 20-year survival probabilities.

The validation of this nomogram is comprehensively presented in Figure 3B. Within the train dataset (Figure 3B1), the Area Under the Curve (AUC) demonstrated strong performance: 0.86 (95 % CI: 0.77–0.94) for the 10-year cumulative follow-up and 0.84 (95 % CI: 0.81–0.87) for the 20-year cumulative follow-up. Similarly, in the independent test dataset (Figure 3B2), the AUC was 0.77 (95 % CI: 0.64–0.91) for the 10-year follow-up and an impressive 0.86 (95 % CI: 0.83–0.90) for the 20-year follow-up.

Overall, these AUC values indicate that our predictive models exhibited good performance in both the train and test datasets, generally achieving an AUC greater than 0.8. While the 10-year follow-up in the testing dataset showed an AUC of 0.77, it remains within the commonly accepted range of 0.70–0.80, which is typically considered acceptable for predictive models [21,22].

Supplementary Figure 1 depicts a further assessment of the nomogram’s predictive accuracy. The x-axis represents the predicted survival probabilities from the final LASSO-Cox model, grouped into three intervals (in tertiles), while the y-axis shows the observed survival probabilities, estimated using the Kaplan-Meier method. Each data point illustrates the comparison between these predicted probabilities and their corresponding observed outcomes. Vertical bars represent the 95 % confidence intervals for the observed probabilities, derived from 1000 bootstrap resamples to ensure robust predictions. The 45-degree diagonal line signifies perfect calibration, with data points closer to this line indicating superior predictive performance. Calibration of 10-year (Supplementary Figure 1A) and 20-year (Supplementary Figure 1B) survival probabilities showed a satisfactory agreement between predicted and observed outcomes in both the training and test datasets.

### Classification of participants at different risk levels

**Fig. 4.**
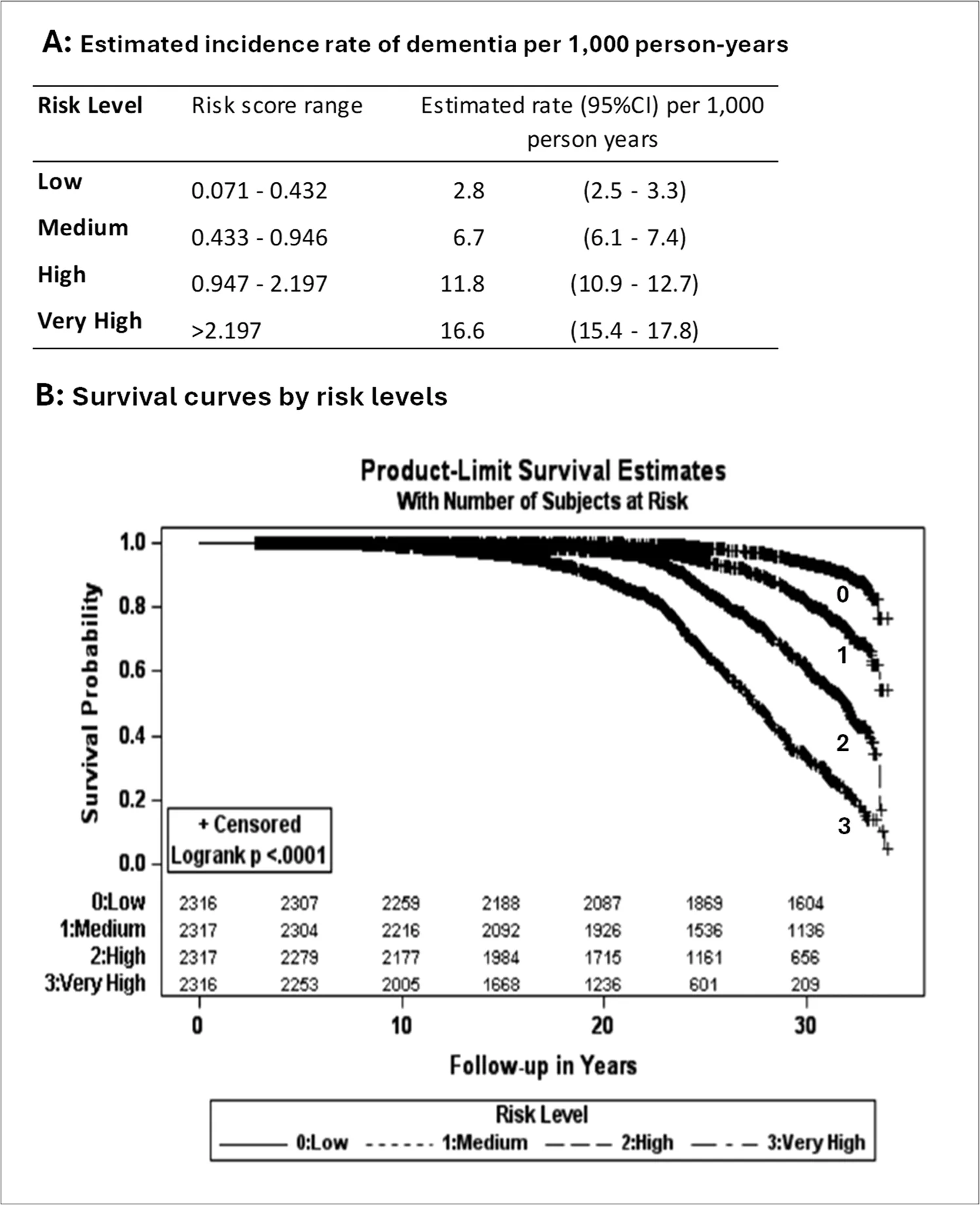
Predicting Dementia Risk. (A) Estimated Incidence Rate Based on LASSO-Cox Risk Scores. (B) Survival curves by four risk levels.

Participants were classified into four distinct risk levels for dementia development based on quartiles (Q) of their LASSO-Cox risk scores: Low risk (Q1), Medium risk (Q2), High risk (Q3), Very-high risk (Q4). The estimated dementia risks per 1000 person-years, along with their 95 % confidence intervals (CI), were 16.6 (15.4–17.8), 11.8 (10.9–12.7), 6.7 (6.1–7.4), and 2.8 (2.5–3.3) in very high, high, medium, and low risk groups, respectively (Figure 4A). Figure 4B visually represents the dementia-free survival curves for each of these four risk levels.

Sensitivity analyses (SA) for SA-Elastic (Stability Analysis), we evaluated the robustness of the 12 predictors selected by LASSO λ_1se_, an optimal mixing parameter of α = 0.8 was selected, indicating that a model with predominantly LASSO-like regularization provided the best fit. The resulting cross-validated C-index (0.766) was nearly identical to the original LASSO training C-index (0.752. All 12 predictors remained in the model with consistent effect directions and magnitudes, confirming the stability of the selected variables. Regarding SA-Cox (Predictive Comparison), the differences in discrimination between the full, 71-predictor model and the parsimonious 12-predictors model were negligible (ΔC-index ≈ 0.002–0008, Table 3). The substantial overlap in the 95 % confidence intervals between the two models demonstrates that the LASSO-selected model maintains comparable predictive accuracy to the full model while significantly enhancing model parsimony.

## Discussion

In this large, population-based observational cohort study, we leveraged advanced LASSO-Cox regression analysis techniques to identify 12 crucial midlife factors that predict late-life dementia risk. Our machine learning (ML)-enabled model showed good-to-strong predictive power [21–23]. It achieved a C-index of 0.771 (95 % CI: 0.754–0.787) in the train dataset, signifying robust internal validity. Furthermore, its performance remained strong in the test dataset, with a C-index of 0.783 (95 % CI: 0.760–0.806), confirming its generalizability and accuracy, values well within the range considered good for complex, multifactorial conditions like dementia. This study is among the first to apply ML techniques to develop a dementia risk prediction model in a large U.S. cohort population with the longest follow-up period.

Our research underscores the multifactorial nature of dementia, emphasizing the need for a holistic approach to predictive modeling. We found that a combination of diverse biological systems significantly contributes to dementia risk. Key factors identified include: (1) Aging: As expected, age remains a primary predictor. (2) Cognitive Decline: Notably, subtle cognitive decline in midlife, as measured by the Digit Symbol Substitution Test (DSST) and Digit Word Recognition Test (DWRT), appeared as a top predictor, reinforcing evidence that these deficits can precede a clinical diagnosis by years or even decades. (3) Genetics: The presence of the APOE ε4 allele significantly contributes to dementia risk, consistent with its known involvement in lipid metabolism, amyloid-beta aggregation, and neuroinflammation [28,29]. (4) Cardiometabolic Health: Markers such as elevated HbA1c, brachial blood pressure, Factor VIII, and apolipoprotein B, along with chronic conditions like hypertension and stroke, are strongly associated with increased risk. (5) Inflammation: Elevated levels of C-reactive protein (CRP) and white blood cell (WBC) count indicate the role of inflammation. Our study highlights serum Factor VIII and apolipoprotein B concentrations as significant predictors of dementia. While further research is needed to fully elucidate the underlying mechanisms, existing evidence offers compelling insights. Elevated Factor VIII concentrations have been linked to an increased risk of microthrombi, which can lead to neuronal damage [30]. Similarly, high apolipoprotein B levels are strongly associated with atherosclerosis, tau dysregulation, and endothelial dysfunction [31]. These findings underscore the potential of these two biomarkers in identifying individuals at higher risk for dementia.

Although previous studies have reported associations between individual biomarkers, such as CRP, Factor VIII, inflammatory markers, or apolipoprotein B, and cognitive decline or dementia, these factors have rarely been evaluated together within a single, penalized survival modeling framework. In our analysis, Factor VIII, white blood cell count, and apolipoprotein B emerged as independent predictors of dementia risk after rigorous LASSO-Cox feature selection and elastic net sensitivity testing, suggesting that their contributions are robust even when considered alongside well-established factors (e.g., age, APOE ε4, and cognitive performance tests). The identification of these markers within the same predictive model highlights the relevance of the cardio-metabolic-inflammation axis as an integrated domain influencing dementia risk. By grouping cardiovascular, metabolic, and inflammatory indicators in a unified, data-driven prediction framework, our study advances understanding of potentially modifiable pathways and supports a clinically meaningful, holistic approach to dementia prevention and risk stratification.

In this study, race/ethnicity was associated with dementia risk in univariable analysis but was not retained in the final LASSO-Cox model, suggesting that its effect may be largely accounted for by other variables such as cognitive test scores (DSST and DWRT), socioeconomic status, and health conditions. The observed differences in mean cognitive scores between Black and White participants, with higher scores among White participants, likely reflect non-biological factors. Race is a socially defined construct that does not reflect underlying biology, and these disparities are shaped by a complex interplay of socioeconomic conditions, social determinants of health, and potential measurement bias in cognitive assessments. Although a detailed examination of these factors is beyond the scope of this study, our findings underscore the need for further research to better understand these differences.

The present study has several strengths. First, we utilized data from the ARIC study, which is one of the largest community and population-based cohort studies in the United States. All measurements of the study variables were conducted using standard protocols, which ensured accuracy, reliability, and were informative to other studies. Second, we applied the LASSO-Cox regression model to develop a dementia risk prediction tool using high-dimensional, longitudinal data. The LASSO-Cox approach offers several advantages over traditional Cox regression and alternative machine learning methods. For example, LASSO performs simultaneous variable selection and regularization, which is particularly valuable in high-dimensional settings where the number of predictors may be large relative to the number of events. Unlike traditional Cox models that can suffer from overfitting when too many covariates are included, LASSO imposes a penalty on the absolute size of the regression coefficients, shrinking some to exactly zero and thus producing a more parsimonious model. This improves both model interpretability and generalizability. Compared to methods like XGBoost or random forests, which are powerful but often treated as "black-box" models, LASSO-Cox retains the transparency and interpretability of regression-based approaches, making it more suitable for clinical translation and risk communication. In particular, the proportional hazards structure of the Cox model allows for time-to-event predictions, while the LASSO penalty helps guard against overfitting, even in the presence of multicollinearity or noise. LASSO-Cox is computationally efficient and can be cross-validated to fine-tune the penalty parameter (lambda), thereby optimizing the model’s performance in a data-driven way. These strengths make it a robust and practical choice for developing clinically relevant risk prediction tools in dementia research. In our sensitivity analysis (SA-Cox), we evaluated whether the penalized approach yielded measurable gains in predictive accuracy by comparing the full Cox model (71 predictors) with the sparse LASSO-Cox selected model (12 predictors). Our findings demonstrated that LASSO achieved substantial model simplification, reducing the predictor set by over 80 % (from 71 to 12 predictors), while maintaining nearly identical predictive performance. This strong agreement highlights the utility of the LASSO approach for enhancing model interpretability and practical application. Furthermore, it suggests that variable shrinkage effectively manages correlated predictors even in larger datasets, facilitating a more stable and parsimonious predictive signature without sacrificing clinical discrimination. Last but not least, the developed nomogram translates the statistical model into an actionable and user-friendly tool for individualized risk estimation at 10- and 20-year follow-ups. It enables clinicians to visually calculate a patient’s risk score based on the 12 selected predictors, advancing the goal of personalized precision medicine. Moreover, stratifying individuals using the LASSO-Cox risk scores allows healthcare providers to recommend tailored preventive strategies by classifying individuals into distinct population- and community-based risk categories (low, moderate, high, and very-high risk groups). This group-based risk prediction approach can enhance risk communication at the population level and inform public health strategies aimed at dementia prevention.

While valuable, these results should be interpreted in light of several limitations. First, although the ARIC study provides a rich, longitudinal, and prospective dataset, its observational design limits the ability to draw causal conclusions. Second, the ARIC cohort consists exclusively of non-Hispanic Black and White participants, which narrows generalizability to other racial/ethnic groups, non-U.S. populations, and individuals younger than 45 years at baseline, are as future research should address. Third, in our LASSO analysis, some potential type I error inflation may persist, although we used 10-fold cross-validation for penalty selection to mitigate overfitting and reduce this risk. As noted by Zhao et al. [32], under cross-validation the LASSO-selected set can, with high probability, behave as if fixed rather than repeatedly searched, which helps maintain approximately correct coverage for subsequent inference. Nevertheless, post-selection inference after LASSO is not guaranteed to eliminate bias entirely, particularly in finite samples [33–36]. Further research applying multiple machine-learning models may help confirm or improve model fitting. Fourth, our survival analyses used Kaplan–Meier estimates, which treated competing events such as death before dementia onset as non-informative censoring. This may overestimate the probability of remaining dementia-free and underestimate cumulative incidence. While Kaplan–Meier curves are commonly reported for their simplicity and comparability, future work could apply competing risk methods such as Fine–Gray sub-distribution hazard models for more accurate estimates [37]. Fifth, we lacked an external dataset to validate our findings. Although we explored resources such as the Multi-Ethnic Study of Atherosclerosis, the Whitehall II Study, UK Biobank, the National Alzheimer’s Coordinating Center, and the Dementia Risk Prediction Project, none provided the same set of predictors, matched key baseline characteristics, or used the same outcome definition as ARIC. Finally, given the potential additive effect of the APOE ε4 allele [38], we acknowledge that treating APOE ε4 status as a binary variable (≥1 copy vs. none) may not fully capture the increased risk among APOE ε4 homozygotes. However, the small number of participants with two ε4 alleles limited our ability to model this effect reliably in multivariable-adjusted analyses.

Despite these limitations, our findings provide an important step forward in understanding dementia risk factors. They also highlight future directions, including replication in diverse populations, examination of longitudinal biomarker trajectories, and evaluation of targeted interventions on cardio-metabolic-inflammatory pathways. Finally, we emphasize the potential of emerging machine learning approaches, such as deep learning and ensemble methods, to identify additional, possibly nonlinear or interactive risk factors beyond those captured by LASSO-Cox, further refining dementia risk prediction.

In conclusion, our research shows that a machine learning model, which combines midlife cognitive function, cardiometabolic and inflammatory markers, and APOE ε4 status, can accurately predict dementia risk. These results strongly support the idea that early identification and proactive intervention on modifiable risk factors could profoundly impact the progression of dementia in older adults.

## Supplementary Material

MMC1

MMC2

Supporting information

Supplementary data associated with this article can be found in the online version at doi:10.1016/j.annepidem.2026.01.007.

## Figures and Tables

**Table 1:** Baseline characteristics of participants by incident dementia status.

Baseline characteristics	Dementia status over the follow-up			
	Non-dementia		Dementia	
	(n=7256)		(n = 2010)	
	Mean / %	(SD)	Mean / %	(SD)

Continuous variables, mean (SD)				
Age, year	53.4	(5.57)	56.2	(5.26)
BMI, kg/m^2^	27.3	(5.16)	27.7	(5.22)
SBP, mmHg	119.3	(18.07)	121.6	(17.58)
DBP, mmHg	72.8	(10.81)	73.3	(10.53)
Ankle SBP, mmHg	143.8	(24.22)	147.6	(24.23)
Brachial SBP, mmHg	126.1	(19.83)	128.6	(19.91)
Total cholesterol, mg/dL	212.9	(40.67)	221.0	(41.08)
HDL, mg/dL	37.6	(10.93)	38.3	(10.92)
LDL, mg/dL	136.2	(38.55)	142.8	(38.76)
Triglycerides, mg/dL	122.9	(63.57)	127.8	(64.83)
Apolipoprotein A-I, mg/dL	133.3	(31.45)	135.1	(31.14)
Apolipoprotein B, mg/dL	92.3	(28.37)	96.9	(27.99)
HbAc1, %	5.7	(1.15)	5.7	(1.11)
Glucose, mg/dL	105.6	(33.90)	106.3	(31.70)
Cystatin C, mg/L	0.88	(0.29)	0.88	(0.18)
DSST z-score	0.13	(0.97)	−0.14	(0.98)
DWRT z-core	0.08	(0.97)	−0.09	(1.01)
WFT z-score	0.08	(0.98)	−0.06	(0.99)
Categorical variables, %				
Female sex	56.7		60.5	
Non-Hispanic Black	19.3		23.5	
Education> High School	47.9		40.9	
Health insurance, yes	92.8		91.2	
Current smokers	25.1		19.3	
Current alcohol use	60.0		51.9	
Non-leisure physic activity	31.8		32.9	
Chronic conditions, %				
Hypertension	32.4		38.3	
Diabetes	9.5		9.9	
Coronary heart disease	5.8		4.1	
Stroke	5.2		6.3	

Values are presented as mean (SD) for continuous variables and % for categorical variables. Abbreviations: BMI: body mass index; SBP: Systolic blood pressure; DBP: Diastolic blood pressure; HDL: high-density lipoprotein cholesterol; LDL: low-density lipoprotein cholesterol; HbA1c: hemoglobin A1c. Cognitive Assessments: DSST: Digit Symbol Substitution Test; DWRT: Delayed Word Recall Test; WFT: Word Fluency Test.

**Table 2:** Lasso coefficients and hazards ratios (HR) of dementia associated with Lasso-Cox selected important features.

Lasso selected key features	Lasso-Cox model	
	Lasso Coefficient βs	HR

Age, year	0.662	1.14
DSST z-score	−0.235	0.76
APOE ε4 (yes vs. no)	0.233	1.89
HbA1c, %	0.102	1.13
Brachial SBP, per 10-unit increase	0.040	1.04
Factor VIII, per 10-unit increase	0.030	1.03
DWRT z-score	−0.027	0.90
Hypertension (yes vs. no)	0.014	1.13
Stroke (yes vs. no)	0.011	1.44
CRP, per 10-unit increase	0.011	1.09
WBC, per 10-unit increase	0.007	1.71
Apolipoprotein B per 10-unit increase	0.003	1.02

Lasso β: Lasso coefficient from training set, via 10-fold cross-validation estimation. DSST: Digit Symbol Substitution Test. APOE ε4: Apolipoprotein E ε4. HbA1c: Hemoglobin A1c. DWRT: Delayed Word Recall Test. CRP: C-reactive protein. WBC: White blood cell.

**Table 3:** Comparison in C-index between Cox (full) and LASSO-Cox (selected) models.

			Training Set		Test Set	
Model	Predictors	Type	C-index	(95 %CI)	C-index	(95 %CI)

Cox	71	Full	0.779	(0.763–0.795)	0.781	(0.758–0.804)
LASSO-Cox	12	Selected	0.771	(0.754–0.787)	0.783	(0.760–0.806)
Difference (ΔC-index)	-	-	0.008		−0.002	

C-index: the Harrell’s Concordance Index. The full model demonstrated slightly higher discrimination in the training set (ΔC = 0.008), while the LASSO-Cox model performed marginally better in the test set (ΔC = −0.002). However, the 95% confidence intervals overlap substantially, indicating that the differences in predictive performance are not statistically significant.
