# Supplementary material for: Machine learning-based LASSO-Cox model for dementia prediction: The role of midlife cardiometabolic, inflammatory, and genetic risk factors in a US cohort
Source: Ann Epidemiol. Author manuscript; Available in PMC 2026 Sep 6. (PMC13546736; doi:10.1016/j.annepidem.2026.01.007)
Supplement: Supplementary file 1 [file NIHMS2191514-supplement-MMC1.docx]

Supplementary Table 1 Candidate predictors and the study outcomes in Lasso-Cox model

Study variables	Visit
**Candidate predictors**	
Ankle-brachial index	1
Age, years	1
Alcohol consumption in groups (never, former, current)	1
Ankle blood pressure, mm Hg	1
Anti-hypercholesteremia drug use (y/n)	1
Ant-hypertensive drug use (y/n)	1
Apolipoprotein B, mg/dl	1
Apolipoprotein E (APOE) ε4 (yes/no)	1
Taking aspirin (y/n)	1
Asthma (y/n)	1
Boday mass index (BMI), kg/m^2	1
BMI in categorical (<18.5, 18.5-24.9, 25-29.9, ≥30 kg/m^2)	1
Brachial blood pressure, mm Hg	1
Center ID (dummy variables was used in LASSO model)	1
Coronary heart disease (y/n)	1
Urea nitrogen, mg/dl	1
Creatinine, mg/dl	1
Serum albumin, g/dl	1
Total protein, mg/dl	1
Uric acid, mg/dl	1
Chronic lung disease, (y/n)	1
C-Reactive protein, mg/dl	2
25-hydroxyvitamin D3, [25(OH)D3], ng/ml	1
Diastolic blood pressure (DBP), mm Hg	1
Diabetes (y/n)	1
Digit Symbol Substitution Test (DSST) z-score	2
Delayed Word Recall Test (DWRT) z score	2
Educational attainment (<high school, high school, >high school)	1
Usual ethanol intake in grams/week	1
Coagulation factor VIII (Factor VIII)	1
Glucose, mg/dl	1
Hemoglobin A1c (HbA1c), %	2
High density lipoprotein cholesterol (HDL), mg/dl	1
Activated Partial Thromboplastin Time (APTT), seconds	1
Fibrinogen, mg/dl	1
Coagulation factor VII, %	1
Antithrombin Activity (ATIII), %	1
Protein C, %	1
Von Willebrand factor (VWF), %	1
Heart failure (y/n)	1
Hematocrit, %	1
Hemoglobin, gm/dl	1
Platelet count	1
Hormone use (0,1,2 for never use, sometime use, and never use)	1
Hypertension, (y/n)	1
Insulin in pmol/L	1
Health insurance (y/n)	1
Low density lipoprotein cholesterol (LDL), mg/dl	1
Physical activity (sedentary, lightly active, moderately active, and very active)	1
Total cholesterol, mg/dl	1
Triglycerides, mg/dl	1
Apolipoprotein A-1 (ApoA-1), mg/dl	1
Apolipoprotein A (Apo a), ug/ml	1
Leisure exercise index	1
Peripheral artery disease, (y/n)	1
Race/ethnicity, (African American and White)	1
Systolic blood pressure, mm Hg	1
Sex (women and men)	1
Smoking status (never, former, current)	1
Stroke (y/n)	1
Cystatin C, mg/L	2
Fructosamine, umol/L	2
Percent glycated albumin (GA), %	2
N-terminal pro b-type natriuretic peptide (NT-proBNP), pg/mL	2
parathyroid hormone, pg/mL	2
Serum Albumin, g/dL	2
Free Triiodothyronine (T3), ng/dL	2
Thyroid-stimulating hormone (TSH), mIU/L	2
White blood cell count (WBC)	1
Waist-to-hip ratio	1
Word Fluency Test (WFT) z-score	2
**Outcomes**	
Incident dementia (y/n)	
Follow-up (years)	

V1: Visit 1 in 1987-1989. Visit 2: 1990-1992.
